# Severe Malnutrition Identified by the Controlling Nutritional Status (CONUT) Score Is Associated With Prolonged Intensive Care Unit (ICU) Stay in Pneumonia Complicated With Respiratory Failure Patients Who Underwent Invasive Mechanical Ventilation

**DOI:** 10.1002/jcla.70109

**Published:** 2025-10-14

**Authors:** Zhijuan Zheng, Guixia Peng, Yue Xiao

**Affiliations:** ^1^ Intensive Care Unit Meizhou People's Hospital Meizhou China

**Keywords:** controlled nutritional status, intensive care unit stay, pneumonia, respiratory failure

## Abstract

**Background:**

Mechanical ventilation is an effective method to improve the ventilation of patients with severe pneumonia and respiratory failure. The length of intensive care unit (ICU) stay reflects the treatment effectiveness of patients. This study was to evaluate the relationship between Controlled Nutritional Status (CONUT) score and prolonged ICU stay in patients with pneumonia complicated by respiratory failure who underwent invasive mechanical ventilation.

**Methods:**

1994 patients who underwent invasive mechanical ventilation were retrospectively analyzed. Medical records (age, gender, body mass index, smoking, drinking, hypertension, diabetes mellitus, lung diseases, blood transfusion, and serum albumin, lymphocyte, cholesterol levels) were collected. The threshold for prolonged ICU stay was defined based on the third quartile (75th percentile) of length of ICU stay. The relationship between CONUT and prolonged ICU stay was analyzed.

**Results:**

The mean ICU stay of patients was 6.6 (3.9, 11.6) days; there were 1495 (75.0%) patients without prolonged ICU stay (< 11.6 days) and 499 (25.0%) with prolonged ICU stay (≥ 11.6 days). The proportion of CONUT severe grade in patients with prolonged ICU stay was higher than that in patients without prolonged ICU stay. Logistic regression analysis showed that CONUT severe grade (odds ratio (OR): 1.298, 95% confidence interval (CI): 1.024–1.646, *p* = 0.031), smoking (OR: 1.475, 95% CI: 1.105–1.968, *p* = 0.008), and blood transfusion (OR: 2.981, 95% CI: 2.406–3.694, *p* < 0.001) were independently associated with prolonged ICU stay.

**Conclusions:**

CONUT severe grade, smoking, and blood transfusion were independently associated with prolonged ICU stay in pneumonia with respiratory failure patients who underwent invasive mechanical ventilation.

## Graphical abstract

This study was to evaluate the relationship between Controlled Nutritional Status(CONUT) score and prolonged ICU stay in patients with pneumonia complicated by respiratory failure who underwent invasive mechanical ventilation. CONUT severe grade, history of smoking, hypertension, and blood transfusion were independently associated with prolonged ICU stay in patients with pneumonia complicated by respiratory failure who underwent invasive mechanical ventilation. It provides a method for evaluating the nutritional status of patients and predicting the prognosis.

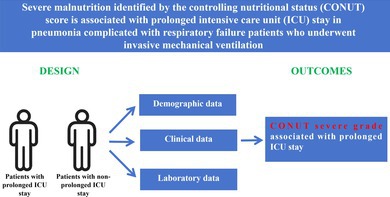

## 1 Introduction

As an acute respiratory tract infection affecting the lungs, pneumonia is mainly characterized by cough, phlegm, and fever [1]. Severe pneumonia patients with lung tissue damage experience limited respiratory function limited, resulting in oxygen supply and demand imbalance, carbon dioxide retention, and other symptoms that can lead to respiratory failure, affecting the prognosis [2, 3]. Respiratory failure is a respiratory dysfunction caused by abnormal lung ventilation function due to various reasons inside and outside the lung, so that gas exchange cannot be effectively carried out [4]. Respiratory failure is a common complication of pneumonia [5] can lead to systemic inflammatory response in patients [6], and in severe cases, it can induce systemic blood circulation disturbance and increase the risk of death [7].

Pneumonia complicated with respiratory failure patients have complex conditions, intense body stress responses, and often involve multiple organ functions [8]. The clinical treatment of such patients is usually anti‐infection and active oxygen therapy, and mechanical ventilation is mainly one [9, 10, 11]. Invasive mechanical ventilation is a life‐supporting technique that involves establishing an artificial airway (such as an oral tracheal tube, nasal tracheal tube, or tracheotomy), connecting the patient to a ventilator, and having the ventilator replace or assist the patient's breathing [12]. Its core function is to maintain effective gas exchange in the patient, correct hypoxemia, alleviate respiratory muscle fatigue, and provide time for the treatment of the underlying disease [12]. The length of intensive care unit (ICU) stay is often used as an indicator of treatment effectiveness in critically ill patients. Prolonged ICU stay leads to an increase in the consumption of medical resources, and may result in higher infection risk, aggravated multi‐organ dysfunction, and other adverse consequences, seriously affecting the prognosis and quality of life of patients [13]. The screening of risk factors for prolonged ICU stay in patients with pneumonia and respiratory failure who underwent invasive mechanical ventilation is of great significance for the prediction of prolonged ICU stay and optimization of treatment.

The nutritional status of the host plays a crucial role in the prognosis of the disease, and malnourished individuals are more susceptible to the effects of the disease and increase the risk of poor prognosis [14]. Currently, the commonly used nutritional assessment tools in clinical practice include the Subjective Global Assessment (SGA) [15] and the Nutritional Risk Screening 2002 (NRS 2002) [16]. However, these tools mostly rely on subjective judgments or require the integration of complex clinical parameters, which limits their convenience and objectivity in the application for ICU patients. Controlled Nutritional Status (CONUT) score is a composite index that combines serum albumin concentration, total lymphocyte count, and total cholesterol concentration to assess an individual's nutritional status [17]. Lymphocyte count, total cholesterol, and serum albumin in CONUT score reflect immune defense function, calorie consumption capacity, and protein reserve capacity, respectively, and CONUT is a reliable indicator to evaluate the nutritional status of individuals [18]. CONUT score has been shown to have potential predictive and evaluative value in the occurrence and prognosis of some lung diseases, such as non‐small cell lung cancer (NSCLC), idiopathic pulmonary fibrosis (IPF), coronavirus disease 2019 (COVID‐19) infection, and pulmonary tuberculosis (PTB) [19, 20, 21, 22]. The objective of this study was to evaluate the relationship between CONUT score and prolonged ICU stay in patients with pneumonia complicated with respiratory failure who underwent invasive mechanical ventilation.

## 2 Materials and Methods

### 2.1 Study Cohort

This study retrospectively analyzed 1994 pneumonia and respiratory failure patients who underwent invasive mechanical ventilation from the Meizhou People's Hospital, from August 2019 to November 2023. The inclusion criteria for patients were as follows: (1) age ≥ 18 years old; (2) patients diagnosed with pneumonia and respiratory failure; (3) underwent invasive mechanical ventilation; and (4) had complete clinical data and laboratory test results. The exclusion criteria for patients were as follows: (1) primary cardiopulmonary dysfunction; (2) combined with systemic infection, severe physical dysfunction, or serious organ dysfunction (such as heart, liver and kidney); (3) combined with autoimmune disease, metastatic diseases, or hematologic malignancy; and (4) clinical records incomplete. This study was approved by the Human Ethics Committees of the Meizhou People's Hospital.

### 2.2 Data Collection

The clinical data were collected, such as age, gender, body mass index (BMI), history of smoking, history of alcohol drinking, hypertension, diabetes mellitus, history of lung diseases, blood transfusion, and laboratory test results (serum albumin, peripheral lymphocyte count, and total cholesterol levels). According to the Chinese standards, BMI is divided into three grades: < 18.5 kg/m^2^ (underweight), 18.5–23.9 kg/m^2^ (normal weight), and ≥ 24.0 kg/m^2^ (overweight) [23, 24]. Blood test data were collected during the first hospital examination. The threshold for prolonged ICU stay was defined based on the third quartile (75th percentile) of the length of ICU stay in all patients.

Pneumonia refers to the inflammation of the terminal airways, alveoli, and interstitial lung tissue, which can be caused by various factors such as pathogenic microorganisms, physical and chemical factors, immune injury, allergy, and drugs. In this study, the diagnosis of pneumonia was based on the Chinese Guidelines for the diagnosis and treatment of pneumonia [25], as follows: (1) clinical symptoms: the patient presents with at least two of the symptoms, fever or hypothermia, cough, expectoration, chest pain, and breathing difficulties; (2) signs: wet rales can be heard during lung auscultation, or there may be an increased respiratory rate (≥ 22 breaths/min) or a faster pulse rate (≥ 100 beats/min); (3) imaging examination: chest X‐ray or CT examination shows new pulmonary infiltrates or consolidation; and (4) laboratory tests: peripheral blood white blood cell count > 10 × 10^9^/L or < 4 × 10^9^/L, accompanied or not by nuclear shift to the left; inflammatory indicators such as C‐reactive protein (CRP) and procalcitonin (PCT) are elevated.

If at least one of the above clinical symptoms and signs is met, combined with the results of imaging examination, and excluding other diseases that can cause pulmonary infiltrates, such as tuberculosis, lung tumors, atelectasis, and pulmonary edema, pneumonia can be diagnosed.

### 2.3 Data Processing and Statistical Analysis

CONUT score is calculated according to serum albumin, peripheral lymphocyte count, and total cholesterol levels:

(1) the serum albumin concentration: ≥ 35 g/L (0 point), 30–34.9 g/L (2 points), 25–29.9 g/L (4 points), and < 25 g/L (6 points); (2) peripheral lymphocyte count: ≥ 1.6 × 10^9^ count/L (0 point), 1.2–1.59 × 10^9^ count/L (1 point), 0.8–1.19 × 10^9^ count/L (2 points), and < 0.8 × 10^9^ count/L (3 points); and (3) total cholesterol level: > 180 mg/dL (0 point), 140–180 mg/dL (1 point), ≥ 100 and < 140 mg/dL (2 points), and < 100 mg/dL (3 points).

CONUT score is assessed as normal when score of 0–1, light when score of 2–4, moderate when score of 5–8, and severe when score of 9–12 [26]. The definition of CONUT shows in Table 1.

**TABLE 1:** Definition of CONUT.

Parameters	Different levels of each parameter and the corresponding score			
Serum albumin (g/L)	≥ 35	30–34.9	25–29.9	< 25
Albumin score	0	2	4	6
Peripheral lymphocyte count (count/L)	≥ 1.6 × 10^9^	1.2–1.59 × 10^9^	0.8–1.19 × 10^9^	< 0.8 × 10^9^
Lymphocyte count score	0	1	2	3
Total cholesterol (mg/dL)	> 180	140–180	≥ 100 and < 140	< 100
Total cholesterol score	0	1	2	3
CONUT score (total)	0–1	2–4	5–8	9–12
Assessment	Normal	Light	Moderate	Severe

Abbreviation: CONUT, controlling nutritional status.

Data analysis was performed using SPSS statistical software version 26.0 (IBM Inc., USA). All continuous variables were subjected to a normality test. Comparisons among variables that follow a normal distribution were analyzed using an independent sample t‐test or one‐way analysis of variance (ANOVA). Variables that do not follow a normal distribution are conducted using the Mann–Whitney *U* test for group comparisons or correlation analysis. Categorical variables were expressed as the number of cases (%), and compared between groups using the *χ* ^2^ test. Logistic regression analysis was used to analyze the relationship of CONUT and prolonged ICU stay adjusting for influencing factors (age, gender, BMI, history of smoking, history of alcohol drinking, hypertension, diabetes mellitus, history of lung diseases, and blood transfusion). *p* < 0.05.

## 3 Results

### 3.1 Comparison of the Clinical Characteristics of Patients With and Without Prolonged ICU Stay

There were 1470 (73.7%) patients who were male and 524 (26.3%) were female; and 660 (33.1%) patients were < 65 years old and 1334 (66.9%) were ≥ 65 years old. About half of the patients (48.8%) had normal weight, and 15.8% of patients were underweight. The proportions of patients with a history of smoking, a history of alcohol drinking, hypertension, diabetes mellitus, and a history of lung diseases were 19.7% (393/1994), 5.3% (106/1994), 48.6% (969/1994), 29.1% (581/1994), and 12.3% (246/1994), respectively. There were 112 (5.6%), 456 (22.9%), 918 (46.0%), and 508 (25.5%) patients with CONUT normal, light, moderate, and severe grade (Table 2).

**TABLE 2:** Comparison of the clinical features between prolonged ICU stay and non‐prolonged ICU stay in patients with pneumonia complicated with respiratory failure who underwent invasive mechanical ventilation.

Clinical characteristics	Total (*n* = 1994)	Non‐prolonged ICU stay (*n* = 1495)	Prolonged ICU stay (*n* = 499)	*p* (*χ* ^2^/*Z*)
Age (years)				
65, *n* (%)	660 (33.1%)	498 (33.3%)	162 (32.5%)	0.742 (*χ* ^2^ = 0.121)
≥ 65, *n* (%)	1334 (66.9%)	997 (66.7%)	337 (67.5%)	
Gender				
Male, *n* (%)	1470 (73.7%)	1101 (73.6%)	369 (73.9%)	0.907 (*χ* ^2^ = 0.018)
Female, *n* (%)	524 (26.3%)	394 (26.4%)	130 (26.1%)	
BMI (kg/m^2^)				
< 18.5, *n* (%)	316 (15.8%)	243 (16.3%)	73 (14.6%)	0.691 (*χ* ^2^ = 0.742)
18.5–23.9, *n* (%)	974 (48.8%)	727 (48.6%)	247 (49.5%)	
≥ 24.0, *n* (%)	704 (35.3%)	525 (35.1%)	179 (35.9%)	
History of smoking				
No, *n* (%)	1601 (80.3%)	1210 (80.9%)	391 (78.4%)	0.217 (*χ* ^2^ = 1.573)
Yes, *n* (%)	393 (19.7%)	285 (19.1%)	108 (21.6%)	
History of alcohol drinking				
No, *n* (%)	1888 (94.7%)	1415 (94.6%)	473 (94.8%)	0.909 (*χ* ^2^ = 0.015)
Yes, *n* (%)	106 (5.3%)	80 (5.4%)	26 (5.2%)	
Hypertension				
No, *n* (%)	1025 (51.4%)	780 (52.2%)	245 (49.1%)	0.235 (*χ* ^2^ = 1.417)
Yes, *n* (%)	969 (48.6%)	715 (47.8%)	254 (50.9%)	
Diabetes mellitus				
No, *n* (%)	1413 (70.9%)	1058 (70.8%)	355 (71.1%)	0.909 (*χ* ^2^ = 0.025)
Yes, *n* (%)	581 (29.1%)	437 (29.2%)	144 (28.9%)	
History of lung diseases				
No, *n* (%)	1748 (87.7%)	1310 (87.6%)	438 (87.8%)	0.938 (*χ* ^2^ = 0.008)
Yes, *n* (%)	246 (12.3%)	185 (12.4%)	61 (12.2%)	
Blood gas analysis				
pH, median (P25, P75)	7.40 (7.35, 7.45)	7.40 (7.35, 7.44)	7.40 (7.34, 7.45)	0.655 (*Z* = −0.446)
PaO_2_ (mmHg), median (P25, P75)	93.30 (67.90, 149.50)	93.80 (67.90, 154.50)	91.60 (68.30, 137.00)	0.346 (*Z* = −0.943)
PaCO_2_ (mmHg), median (P25, P75)	32.90 (27.30, 40.40)	33.10 (27.30, 40.40)	31.80 (27.50, 40.00)	0.285 (*Z* = −1.070)
CONUT				
Normal, *n* (%)	112 (5.6%)	81 (5.4%)	31 (6.2%)	0.006 (*χ* ^2^ = 12.275)
Light, *n* (%)	456 (22.9%)	353 (23.6%)	103 (20.6%)	
Moderate, *n* (%)	918 (46.0%)	708 (47.4%)	210 (42.1%)	
Severe, *n* (%)	508 (25.5%)	353 (23.6%)	155 (31.1%)	
Blood transfusion				
No, *n* (%)	1267 (63.5%)	1045 (69.9%)	222 (44.5%)	< 0.001 (*χ* ^2^ = 104.277)
Yes, *n* (%)	727 (36.5%)	450 (30.1%)	277 (55.5%)	
ICU stay (days)	6.6 (3.9, 11.6)			

Abbreviations: BMI, body mass index; CONUT, controlling nutritional status; ICU, Intensive Care Unit; p25, 25th percentile; p75, 75th percentile.

In this study, the mean ICU stay of patients was 6.6 (3.9, 11.6) days. The threshold for prolonged ICU stay was defined as ≥ 11.6 days. There were 1495 (75.0%) patients without prolonged ICU stay (< 11.6 days) and 499 (25.0%) patients with prolonged ICU stay (≥ 11.6 days). The proportion of patients with prolonged ICU stay who received blood transfusion treatment (55.5% vs. 30.1%, *p* < 0.001) was higher than that of those without prolonged ICU stay. There were significant differences in the distribution of CONUT grade (*p =* 0.006) between patients with and without prolonged ICU stay. The proportion of CONUT severe grade in patients with prolonged ICU stay was higher than in patients without prolonged ICU stay. There was no statistically significant difference in age, gender, and BMI distribution and proportion of history of smoking, alcohol drinking, hypertension, diabetes mellitus, and history of lung diseases between the prolonged ICU stay and non‐prolonged ICU groups (Table 2).

### 3.2 Comparison of the Clinical Characteristics of Patients With and Without Prolonged ICU Stay in Patients Treated With Blood Transfusion

In patients treated with blood transfusion (*n* = 727), there were 450 patients without prolonged ICU stay and 277 patients with prolonged ICU stay. The proportion of patients with prolonged ICU stay who had a history of alcohol drinking (3.2% vs. 7.1%, *p* = 0.031) was lower, and the proportion of hypertension (52.0% vs. 44.0%, *p* = 0.039) was higher than those without prolonged ICU stay, respectively. There was no difference in the distribution of CONUT grade between patients with and without prolonged ICU stay (Table 3).

**TABLE 3:** Comparison of the clinical characteristics of patients with prolonged ICU stay and non‐prolonged ICU stay in patients treated with blood transfusion.

Clinical characteristics	Blood transfusion (*n* = 727)	Non‐prolonged ICU stay (*n* = 450)	Prolonged ICU stay (*n* = 277)	*p* (*χ* ^2^/*Z*)
Age (years)				
65, *n* (%)	262 (36.0%)	171 (38.0%)	91 (32.9%)	0.176 (*χ* ^2^ = 1.971)
≥ 65, *n* (%)	465 (64.0%)	279 (62.0%)	186 (67.1%)	
Gender				
Male, *n* (%)	518 (71.3%)	312 (69.3%)	206 (74.4%)	0.152 (*χ* ^2^ = 2.122)
Female, *n* (%)	209 (28.7%)	138 (30.7%)	71 (25.6%)	
BMI (kg/m^2^)				
< 18.5, *n* (%)	136 (18.7%)	96 (21.3%)	40 (14.4%)	0.040 (*χ* ^2^ = 6.447)
18.5–23.9, *n* (%)	354 (48.7%)	206 (45.8%)	148 (53.4%)	
≥ 24.0, *n* (%)	237 (32.6%)	148 (32.9%)	89 (32.1%)	
History of smoking				
No, *n* (%)	619 (85.1%)	390 (86.7%)	229 (82.7%)	0.163 (*χ* ^2^ = 2.164)
Yes, *n* (%)	108 (14.9%)	60 (13.3%)	48 (17.3%)	
History of alcohol drinking				
No, *n* (%)	686 (94.4%)	418 (92.9%)	268 (96.8%)	0.031 (*χ* ^2^ = 4.806)
Yes, *n* (%)	41 (5.6%)	32 (7.1%)	9 (3.2%)	
Hypertension				
No, *n* (%)	385 (53.0%)	252 (56.0%)	133 (48.0%)	0.039 (*χ* ^2^ = 4.389)
Yes, *n* (%)	342 (47.0%)	198 (44.0%)	144 (52.0%)	
Diabetes mellitus				
No, *n* (%)	508 (69.9%)	313 (69.6%)	195 (70.4%)	0.868 (*χ* ^2^ = 0.058)
Yes, *n* (%)	219 (30.1%)	137 (30.4%)	82 (29.6%)	
History of lung diseases				
No, *n* (%)	649 (89.3%)	405 (90.0%)	244 (88.1%)	0.459 (*χ* ^2^ = 0.655)
Yes, *n* (%)	78 (10.7%)	45 (10.0%)	33 (11.9%)	
Blood gas analysis				
pH, median (P25, P75)	7.40 (7.34, 7.44)	7.40 (7.33, 7.44)	7.40 (7.35, 7.44)	0.656 (*Z* = −0.446)
PaO_2_ (mmHg), median (P25, P75)	93.70 (68.20, 154.60)	93.80 (68.48, 158.35)	93.30 (66.30, 143.05)	0.455 (*Z* = −0.747)
PaCO_2_ (mmHg), median (P25, P75)	30.80 (26.10, 37.40)	30.35 (25.70, 37.20)	31.20 (27.05, 37.85)	0.159 (*Z* = −1.410)
CONUT				
Normal, *n* (%)	26 (3.6%)	18 (4.0%)	8 (2.9%)	0.300 (*χ* ^2^ = 3.686)
Light, *n* (%)	116 (16.0%)	70 (15.6%)	46 (16.6%)	
Moderate, *n* (%)	341 (46.9%)	221 (49.1%)	120 (43.3%)	
Severe, *n* (%)	244 (33.6%)	141 (31.3%)	103 (37.2%)	

Abbreviations: BMI, body mass index; CONUT, controlling nutritional status; ICU, Intensive Care Unit; p25, 25th percentile; p75, 75th percentile.

### 3.3 Comparison of the Clinical Characteristics of Patients With Prolonged ICU Stay and Non‐Prolonged ICU Stay in Patients Treated Without Blood Transfusion

In patients treated without blood transfusion (*n* = 1267), there were 1045 (82.5%) patients with non‐prolonged ICU stay and 222 (17.5%) patients with prolonged ICU stay. There was no significant difference in age, gender, BMI, CONUT grade, smoking history, drinking history, hypertension, diabetes mellitus, and pulmonary disease history between the two groups (Table 4).

**TABLE 4:** Comparison of the clinical characteristics of patients with prolonged ICU stay and non‐prolonged ICU stay in patients treated without blood transfusion.

Clinical characteristics	Non‐blood transfusion (*n* = 1267)	Non‐prolonged ICU stay (*n* = 1045)	Prolonged ICU stay (*n* = 222)	*p* (*χ* ^2^/*Z*)
Age (years)				
65, *n* (%)	398 (31.4%)	327 (31.3%)	71 (32.0%)	0.874 (*χ* ^2^ = 0.040)
≥ 65, *n* (%)	869 (68.6%)	718 (68.7%)	151 (68.0%)	
Gender				
Male, *n* (%)	952 (75.1%)	789 (75.5%)	163 (73.4%)	0.550 (*χ* ^2^ = 0.424)
Female, *n* (%)	315 (24.9%)	256 (24.5%)	59 (26.6%)	
BMI (kg/m^2^)				
< 18.5, *n* (%)	180 (14.2%)	147 (14.1%)	33 (14.9%)	0.346 (*χ* ^2^ = 2.108)
18.5–23.9, *n* (%)	620 (48.9%)	521 (49.9%)	99 (44.6%)	
≥ 24.0, *n* (%)	467 (36.9%)	377 (36.1%)	90 (40.5%)	
History of smoking				
No, *n* (%)	982 (77.5%)	820 (78.5%)	162 (73.0%)	0.077 (*χ* ^2^ = 3.172)
Yes, *n* (%)	285 (22.5%)	225 (21.5%)	60 (27.0%)	
History of alcohol drinking				
No, *n* (%)	1202 (94.9%)	997 (95.4%)	205 (92.3%)	0.066 (*χ* ^2^ = 3.533)
Yes, *n* (%)	65 (5.1%)	48 (4.6%)	17 (7.7%)	
Hypertension				
No, *n* (%)	640 (50.5%)	528 (50.5%)	112 (50.5%)	1.000 (*χ* ^2^ < 0.001)
Yes, *n* (%)	627 (49.5%)	517 (49.5%)	110 (49.5%)	
Diabetes mellitus				
No, *n* (%)	905 (71.4%)	745 (71.3%)	160 (72.1%)	0.870 (*χ* ^2^ = 0.055)
Yes, *n* (%)	362 (28.6%)	300 (28.7%)	62 (27.9%)	
History of lung diseases				
No, *n* (%)	1099 (86.7%)	905 (86.6%)	194 (87.4%)	0.828 (*χ* ^2^ = 0.098)
Yes, *n* (%)	168 (13.3%)	140 (13.4%)	28 (12.6%)	
Blood gas analysis				
pH, median (P25, P75)	7.40 (7.35, 7.45)	7.40 (7.35, 7.44)	7.41 (7.33, 7.45)	0.466 (*Z* = −0.729)
PaO_2_ (mmHg), median (P25, P75)	93.10 (67.80, 146.40)	93.80 (67.55, 149.00)	90.90 (69.95, 133.00)	0.424 (*Z* = −0.800)
PaCO_2_ (mmHg), median (P25, P75)	33.90 (28.50, 42.50)	34.00 (28.60, 42.35)	33.45 (28.38, 43.03)	0.683 (*Z* = −0.408)
CONUT				
Normal, *n* (%)	86 (6.8%)	63 (6.0%)	23 (10.4%)	0.056 (*χ* ^2^ = 7.538)
Light, *n* (%)	340 (26.8%)	283 (27.1%)	57 (25.7%)	
Moderate, *n* (%)	577 (45.5%)	487 (46.6%)	90 (40.5%)	
Severe, *n* (%)	264 (20.8%)	212 (20.3%)	52 (23.4%)	

Abbreviations: BMI, body mass index; CONUT, controlling nutritional status; ICU, Intensive Care Unit; p25, 25th percentile; p75, 75th percentile.

### 3.4 Logistic Regression Analysis of Risk Factors of Prolonged ICU Stay

The results of univariate analysis indicated that CONUT severe grade (odds ratio (OR): 1.458, 95% confidence interval (CI): 1.165–1.824, *p* = 0.001), and blood transfusion (OR: 2.898, 95% CI: 2.353–3.569, *p* < 0.001) were significantly associated with prolonged ICU stay (Table 4).

In multivariate logistic regression analysis, CONUT severe grade (OR: 1.298, 95% CI: 1.024–1.646, *p* = 0.031), history of smoking (OR: 1.475, 95% CI: 1.105–1.968, *p* = 0.008), and blood transfusion (OR: 2.981, 95% CI: 2.406–3.694, *p* < 0.001) were independently associated with prolonged ICU stay (Table 5).

**TABLE 5:** Logistic regression analysis of risk factors associated with prolonged ICU stay.

Variables	Unadjusted values		Adjusted values	
	OR (95% CI)	*p*	Adjusted OR (95% CI)	*p*
CONUT (Severe vs. non‐severe)	1.458 (1.165–1.824)	0.001	1.298 (1.024–1.646)	0.031
Age (≥ 65 vs. < 65, years)	1.039 (0.837–1.290)	0.728	1.056 (0.839–1.329)	0.641
Gender (male vs. female)	1.016 (0.807–1.279)	0.894	0.971 (0.756–1.247)	0.817
BMI (kg/m^2^)				
18.5–23.9	1.000 (reference)	—	1.000 (reference)	—
< 18.5	0.884 (0.656–1.192)	0.419	0.793 (0.580–1.085)	0.147
≥ 24.0	1.004 (0.803–1.254)	0.975	1.067 (0.844–1.349)	0.587
History of smoking (yes vs. no)	1.173 (0.914–1.504)	0.210	1.475 (1.105–1.968)	0.008
History of alcohol drinking (yes vs. no)	0.972 (0.617–1.532)	0.903	0.797 (0.481–1.321)	0.379
Hypertension (yes vs. no)	1.131 (0.923–1.385)	0.234	1.162 (0.930–1.452)	0.187
Diabetes mellitus (yes vs. no)	0.982 (0.785–1.228)	0.874	0.911 (0.718–1.156)	0.444
History of lung diseases (yes vs. no)	0.986 (0.724–1.343)	0.930	1.091 (0.789–1.507)	0.599
Blood transfusion (yes vs. no)	2.898 (2.353–3.569)	< 0.001	2.981 (2.406–3.694)	< 0.001

Abbreviations: BMI, body mass index; CI, confidence interval; CONUT, controlling nutritional status; ICU, Intensive Care Unit; OR, odds ratio.

**FIGURE 1:**
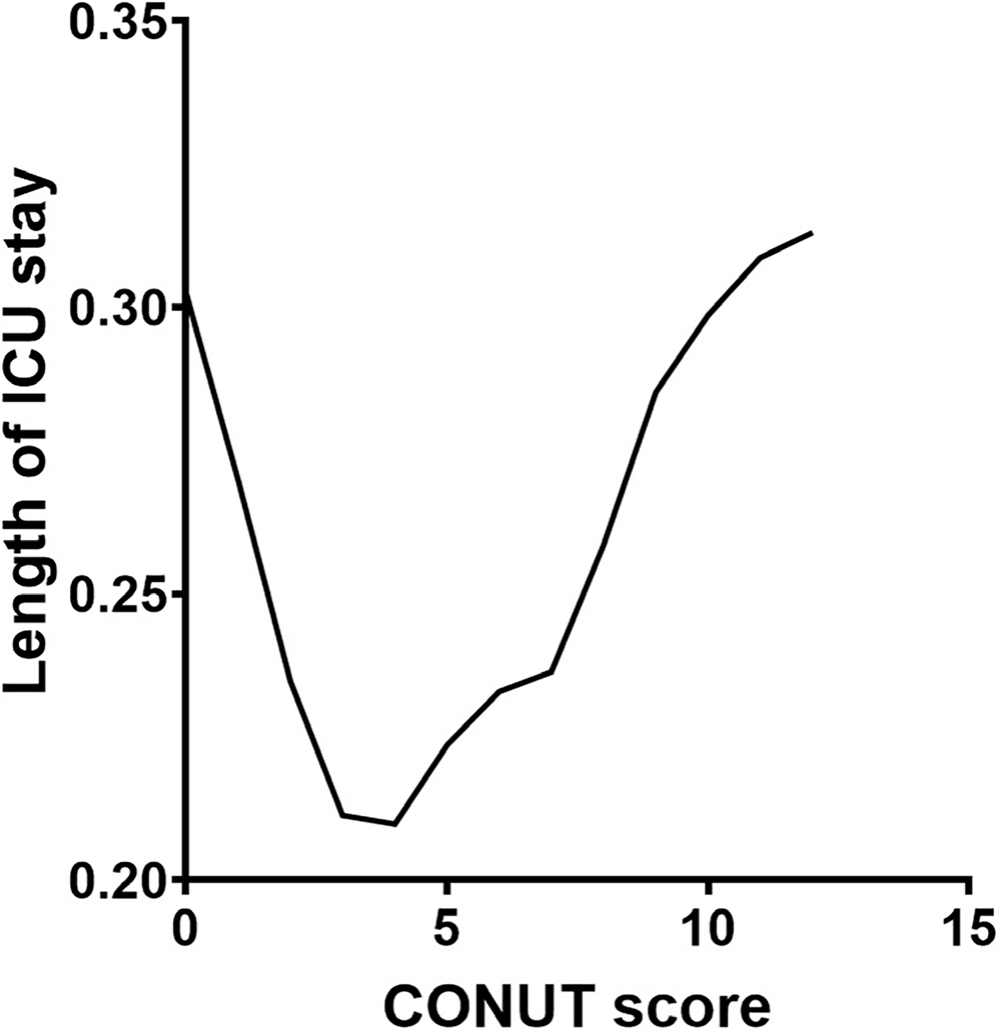
Smoothed curves of the associations between CONUT and the incidence of prolonged ICU stay in pneumonia complicated with respiratory failure patients who underwent invasive mechanical ventilation.

To further explore the relationship between CONUT and the incidence of prolonged ICU stay of patients, we used a smooth curve to determine the potential relationship within the correlation (Figure 1). The relationship between CONUT and the prolonged ICU stay of patients showed this trend: when CONUT was < 4, for every 1 unit increase in CONUT, the incidence of prolonged ICU stay increased by 0.853 times (*p* = 0.184); conversely, when CONUT was ≥ 4, the incidence of prolonged ICU stay increased by 1.154 times (*p* = 0.044) (Table 6).

**TABLE 6:** Analyses of CONUT on prolonged ICU stay for patients with pneumonia complicated with respiratory failure who underwent invasive mechanical ventilation.

	Crude *β*/OR (95% CI)	*p*	Adjusted *β*/OR (95% CI)	*p*
Prolonged ICU stay				
CONUT 0–3	0.845 (0.676–1.057)	0.140	0.853 (0.675–1.09)	0.184
CONUT 4–12	1.169 (1.019–1.322)	0.007	1.154 (1.109–1.319)	0.044

*Note:* Adjust for: age, gender, BMI, history of smoking, history of alcohol drinking, hypertension, diabetes mellitus, history of lung diseases, and blood transfusion.

Abbreviations: CONUT, controlling nutritional status; ICU, Intensive Care Unit.

## 4 Discussion

The key to the treatment of pneumonia combined with respiratory failure is to rapidly improve hypoxia, restore respiratory function, and improve dysfunction [27]. Invasive mechanical ventilation is an effective way to rapidly improve hypoxia in patients, which can improve ventilation status and alleviate symptoms [28, 29]. The prognosis of pneumonia and respiratory failure patients is considered to be related to multiple factors, and nutritional risk is considered to be a high‐risk factor [30, 31]. The purpose of this study was to investigate whether nutritional status assessed based on the CONUT score was associated with prolonged ICU stay in patients with pneumonia in combination with respiratory failure who underwent invasive mechanical ventilation. The results show that CONUT severe grade, history of smoking, and blood transfusion were independently associated with prolonged ICU stay in patients with pneumonia complicated with respiratory failure who underwent invasive mechanical ventilation.

Nutritional status assessment is a crucial step in clinical diagnosis and treatment, and its accuracy directly affects the formulation of intervention strategies and the improvement of prognosis [32, 33]. Currently, the most widely used nutritional status assessment methods include prognostic nutritional index (PNI), NRS 2002, and CONUT [34]. PNI relies solely on serum albumin and peripheral blood lymphocyte count, which can reflect protein reserves and immune status, but lacks assessment of energy metabolism (such as lipid reserves) [35]. NRS 2002 is mainly based on weight loss, reduced dietary intake, severity of the disease, and age, focusing on risk screening rather than quantification of nutritional status [36]. CONUT integrates protein, energy, and immune indicators to achieve multi‐dimensional quantification of nutritional status, and can better reflect the balance of protein reserves, energy metabolism, and immune function. In addition, CONUT relies entirely on objective laboratory data, reducing subjective bias and enhancing assessment consistency. Furthermore, the design of CONUT enables it to not only meet the requirements of rapid screening but also support long‐term dynamic monitoring, and it is applicable to a wide range of populations.

In this study, CONUT severe grade was independently associated with prolonged ICU stay in patients with pneumonia complicated by respiratory failure who underwent invasive mechanical ventilation. In the CONUT score, peripheral blood lymphocyte count is an indicator reflecting the immune status of the body. T lymphocytes were associated with treatment decisions and prognosis in patients with respiratory failure [37, 38]. Lower cholesterol levels were associated with more severe acute respiratory distress syndrome in patients [39] and increased mortality in patients with community‐acquired pneumonia (CAP) [40]. Some studies have suggested that serum albumin was associated with mortality in patients with pneumonia [41, 42]. As a composite index combining lymphocyte, total cholesterol, and serum albumin, CONUT can comprehensively reflect the immune nutritional status of patients and may be related to prolonged ICU stay in patients with pneumonia in combination with respiratory failure who underwent invasive mechanical ventilation.

The relationship of CONUT score in pulmonary diseases has been reported. Some studies found that higher levels of malnutrition on the CONUT score were associated with adverse outcomes in patients with spinal tuberculosis [43] and pulmonary tuberculosis [22]. Iwanami Y et al. found that moderate malnutrition based on CONUT score was associated with an increased risk of death in elderly patients with idiopathic pulmonary fibrosis [20]. CONUT was a reliable predictor of pneumonia in patients with cerebral hemorrhage [44]. Lee et al. found that preoperative CONUT score was a predictor of postoperative pulmonary complications in patients with non‐small cell lung cancer [19]. Among COVID‐19 patients, the risk of adverse outcomes was higher in the high‐CONUT group than in the low‐CONUT group [21, 45]. Some studies showed that the more severe the level of malnutrition based on CONUT score, the prolonged hospital stay for COVID‐19 patients [46, 47]. This study was the first attempt to evaluate the prolonged ICU stay of patients with pneumonia and respiratory failure using the CONUT score. The results indicated that CONUT might be a valuable prognostic indicator for this patient population.

In addition, the history of smoking and blood transfusion were associated with prolonged ICU stay in patients with pneumonia combined with respiratory failure who underwent invasive mechanical ventilation in the present study. Some research revealed that those with a history of smoking were more likely to be admitted to the ICU among patients with COVID‐19 infection [48, 49, 50], and community‐acquired pneumonia (CAP) [51]. And smoking was associated with mortality in people with COVID‐19 pneumonia [52]. However, Alarcon‐Calderon et al. found that smoking had no effect on ICU stay and prognosis in patients with respiratory failure due to COVID‐19 pneumonia [53]. It may be due to poor lung function in patients with a history of smoking and the possibility of a poorer prognosis [54]. Thrombocytopenia and anemia can occur in severe pneumonia due to inflammation, bleeding, and disseminated intravascular coagulation [55, 56]. The symptoms of hypoxia in partial patients with tissue hypoxia may be effectively improved after blood transfusion of red blood cells [57, 58]. Blood transfusion can activate the immune system and trigger an early inflammatory immune response [59]. Fluid resuscitation can cause hypothermia, coagulation dysfunction, acidosis, and aggravate tissue damage [59]. The poor prognosis of blood transfusion patients may be related to shortened red blood cell life, inflammatory factor production, and decreased erythropoietic cell production in the bone marrow [60, 61].

There are some limitations in our study. First, the patients included in our study did not represent all ICU pneumonia and respiratory failure patients in China. In fact, different medical institutions are not uniform in terms of case mix, available resources, and quality of care. Second, multivariate analysis was limited by the included variables, and it was unable to eliminate the influence of confounding factors (such as Acute Physiology and Chronic Health Evaluation II (APACHE II) score, Sequential Organ Failure Assessment (SOFA) score) that were not included in the analysis. APACHE II score is a classic indicator for assessing the severity of diseases in critically ill patients; it reflects the degree of acute physiological disorder and chronic health status of the patients and may be related to the length of ICU stay [62]. SOFA score can dynamically assess the degree of organ failure in patients and is an important basis for judging the progression of the disease and prognosis [63]. This study failed to exclude the interactive influence between the APACHE II and SOFA scores and the CONUT score in reflecting severe malnutrition, which may to some extent affect the accuracy and robustness of the conclusion. Third, in this study, the 75th percentile of length of ICU stay was used as the cut‐off value to define prolonged ICU stay. This approach was determined based on previous studies [64, 65, 66, 67, 68]. Currently, in the absence of a unified and standard evaluation criterion for prolonged ICU hospitalization, the research results may vary due to different definition criteria. Finally, due to the lack of detailed data on alternative treatment options and course of treatment for pneumonia and respiratory failure patients, there was a lack of analysis of the impact of treatment data on prolonged ICU stay.

In conclusion, CONUT severe grade, history of smoking, and blood transfusion were independently associated with prolonged ICU stay in patients with pneumonia combined with respiratory failure who received invasive mechanical ventilation. It indicates that in the specific patient group with a history of smoking and pneumonia complicated with respiratory failure and receiving invasive mechanical ventilation, the severe malnutrition status reflected by the CONUT score is an important factor affecting their length of stay in the ICU. However, this study has some limitations, so the results of this research require further studies to be verified. Future research can be conducted in multiple directions. Firstly, more studies should be carried out to reduce bias and more rigorously verify the relationship between the CONUT score and the length of ICU stay. Secondly, include more indicators to improve the evaluation model and more accurately reveal the impact of nutritional status on the prognosis of patients. Furthermore, further exploration of the effects of different nutritional support strategies on improving the prognosis of patients with severe malnutrition can be conducted to provide more powerful evidence for the clinical formulation of personalized nutritional management strategies.

## 5 Conclusions

CONUT severe grade, history of smoking, and blood transfusion were independently associated with prolonged ICU stay in patients with pneumonia complicated by respiratory failure who underwent invasive mechanical ventilation. It provides a method for evaluating the nutritional status of patients and predicting the prognosis.

## Author Contributions

Zhijuan Zheng and Ming Yu designed the study. Zhijuan Zheng, Guixia Peng, and Yue Xiao collected clinical data. Zhijuan Zheng and Ming Yu analyzed the data. Zhijuan Zheng and Ming Yu prepared the manuscript. All authors were responsible for critical revisions, and all authors read and approved the final version of this work.

## Conflicts of Interest

The authors declare no conflicts of interest.

## Data Availability

The data that support the findings of this study are available from the corresponding author upon reasonable request.
